# Identification of novel antiviral host factors by functional gene expression analysis using *in vitro* HBV infection assay systems

**DOI:** 10.1371/journal.pone.0314581

**Published:** 2025-03-06

**Authors:** Takuto Nosaka, Tatsushi Naito, Yu Akazawa, Kazuto Takahashi, Hidetaka Matsuda, Masahiro Ohtani, Tsutomu Nishizawa, Hiroaki Okamoto, Yasunari Nakamoto

**Affiliations:** 1 Second Department of Internal Medicine, Faculty of Medical Sciences, University of Fukui, Fukui, Japan; 2 Division of Virology, Department of Infection and Immunity, Jichi Medical University School of Medicine, Tochigi, Japan; Centers for Disease Control and Prevention, UNITED STATES OF AMERICA

## Abstract

To cure hepatitis B virus (HBV) infection, it is essential to elucidate the function of hepatocyte host factors in regulating the viral life cycle. Signaling and transcription activator of transcription (STAT)1 play important roles in immune responses, but STAT1-independent pathways have also been shown to have important biological reactivity. Using an *in vitro* HBV infection assay system, the current study aimed to investigate the STAT1-independent host factors that contribute to the control of viral infection by comprehensive functional screening. The *in vitro* HBV infection system was established using primary human hepatocytes (PXB cells) infected with HBV derived from a plasmid containing the 1.3-mer HBV genome. Comprehensive functional studies were performed using small interfering RNA (siRNA) and vector transfection and analyzed using microarrays. Knockdown of STAT1 increased viral products in HBV-transfected HepG2 cells, but decreased in HBV-infected PXB cells. RNA microarray was performed using HBV-infected PXB cells with STAT1 knockdown. Fumarylacetoacetate hydrolase (FAH) was extracted by siRNA of genes in PXB cells altered by STAT1 knockdown. Transfection of FAH inhibited HBV replication. Dimethyl fumarate (DMF), the methyl ester of FAH metabolite, showed antiviral effects by inducing autophagy and anti-HBV-related genes. Independently of STAT1, FAH was identified as a host factor that contributes to the control of viral infection, and its metabolite, DMF, exhibited antiviral activity. These results suggest that the novel host factor FAH and its metabolites may be an innovative therapeutic strategy to control the HBV life cycle.

## Introduction

Chronic hepatitis B (CHB), caused by the hepatitis B virus (HBV), is a leading cause of liver fibrosis, cirrhosis, and hepatocellular carcinoma (HCC) worldwide [1,2]. HCC is currently the second leading cause of death from cancer, and more than 50% of HCC cases are linked to HBV infection in the most affected areas [3]. Current treatments for CHB include nucleos(t)ide analogues (NUCs) and pegylated interferon-γ (PEG-IFN-γ) [4]. Although NUCs can significantly suppress HBV DNA, they do not act directly on covalently closed circular DNA (cccDNA), the intranuclear template for HBV replication, and long-term treatment is usually required to maintain HBV suppression. PEG-IFN-γ-based therapies have the potential to cure infections, but suffer from low response rates and severe side effects.

Targeting the hepatocyte host factors involved in the viral life cycle may be a promising therapeutic approach to overcome cccDNA persistence. Among the host factors reported to be associated with the HBV life cycle, the signal transducer and activator of transcription (STAT) 1 protein plays a key role in the immune response by transducing signals from type I–III interferons (IFNs) [5–7]. It has been established that the major biological responses to IFN-γ are gene products regulated by the Janus kinase (JAK)-STAT pathway [8]. However, comprehensive screening, including microarray analysis using STAT1-deficient cells, has revealed that STAT-independent pathways also play an important role in the biological response to IFN-γ [9]. Elucidating the function of host factors associated with the HBV life cycle provides not only a basic understanding of HBV infection, but also has the potential to identify new antiviral targets and facilitate the development of new therapeutic strategies [10].

Using an *in vitro* HBV infection assay system [11, 12], this study was designed to investigate the STAT1-independent anti-HBV mechanisms in the HBV life cycle. Although the antiviral activity of STAT1 was seen in HepG2 cells transfected with the HBV genome, the viral products were decreased by small interfering RNA (siRNA) knockdown experiments of the STAT1 molecule in the infection system. Furthermore, comprehensive functional screening identified fumarylacetoacetate hydrolase (FAH) as a previously unidentified candidate that contributes to the control of viral infection independently of STAT1. Moreover, fumarate, a tyrosine metabolite produced by FAH, was shown to exhibit anti-HBV effects by inducing autophagy and anti-HBV-related genes in hepatocytes.

## Materials and methods

### Cell line

The human liver cancer cell line HepG2 was obtained from the American Type Culture Collection (Manassas, VA, USA) and cultured at 37°C with 5% CO2 and RPMI-1640 (Sigma-Aldrich, St. Louis, MO, USA) containing 10% fetal bovine serum (FBS), 2 mmol/L L-glutamine, 1 μmol/L sodium pyruvate, 0.1 mmol/L nonessential amino acids, and 100 U/mL penicillin/50 μg/mL streptomycin (Gibco, Grand Island, NY, USA) [12].

### Primary human hepatocytes

Primary human hepatocytes (PXB cells) derived from chimeric urokinase-type plasminogen activator/severe combined immunodeficiency (uPA/SCID) mice with humanized livers were purchased from PhoenixBio Co., Ltd. (Hiroshima, Japan) and cultured in BioCoat Collagen I white plates (Corning Life Science, Tewksbury, MA, USA) using maintenance medium, as described previously [11,12].

### HBV plasmid transfection into HepG2 cells and treatment

The 1.3-mer HBV genome [genotype C2, both basal core promoter (BCP) A1762T/G1764A mutation and precore G1896A mutation, accession number AB819615] was inserted into the KpnΙ and NotΙ restriction sites of pBluescript II SK (Agilent Technologies, Santa Clara, USA). Subsequently, the recombinant HBV plasmid was introduced into HepG2 cells through transfection using Lipofectamine LTX Reagent (Thermo Fisher Scientific, Waltham, MA, USA) [12]. HepG2 cells transfected with HBV, exhibiting stable production of hepatitis B surface antigen (HBsAg) in the supernatant, were identified as HepG2.D11 clone. Dimethyl fumarate (DMF, Sigma-Aldrich, 242926) was added to the medium at different concentrations.

### In vitro HBV infection to primary human hepatocyte

HBV was collected from the supernatant of HepG2 cells transfected with HBV plasmids and digested with recombinant DNase I (Takara Bio, Shiga, Japan). HBV DNA was extracted using SMI TEST EX-R&D (Medical & Biological Laboratories Co., Ltd., Nagano, Japan), and the amount was determined using real-time quantitative polymerase chain reaction (qPCR). PXB cells were infected with HBV at a multiplicity of infection (MOI) of 500 for 24 h in the presence of 4% PEG 8000 (Sigma-Aldrich) [12].

### Interferon treatment

IFN-γ (PeproTech, Rocky Hill, NJ, USA) was diluted in culture medium and added to PXB cells and HepG2.D11 cells at the concentrations of 5 ×  10^3^ ng/mL as described previously [11].

### Quantification of HBsAg

The HBsAg levels in the supernatant were determined using Lumipulse HBsAg-HQ immunoassay (Fujirebio Inc., Tokyo, Japan) [12].

### RNA and DNA extraction and cDNA synthesis

Total RNA was extracted using TRIzol reagent (Thermo Fisher Scientific) and cDNA synthesis was performed using a High-Capacity RNA-to-cDNA Kit (Applied Biosystems, Foster City, CA, USA). DNA was extracted from the supernatant and HepG2.D11 and PXB cells using SMITEST EX-R&D (MBL) [12].

### Quantification of HBV DNA and HBV RNA

Two pairs of primers, corresponding to the DNA regions of the HBV genome, were used for the assay (Table 1). Nested PCR was performed for HBV DNA, and the quantitative gene expression levels of HBV DNA and RNA were determined by real-time PCR using the StepOne Plus system (Applied Biosystems) [12]. Primers and probes were obtained from Takara Bio and Applied Biosystems (Table 1). Glyceraldehyde-3-phosphate dehydrogenase (GAPDH; Applied Biosystems) and transferrin receptor (TFRC; Sigma-Aldrich) were used as endogenous controls [12]. The conversion of HBV DNA was performed by the following equation: 1 pg/ml =  2.83 ×  10^5^ copies/ml =  5.45 log10 copies/ml [13,14].

**Table 1:** Nucleotide sequences of primers and a probe used for real-time quantitative polymerase chain reaction.

Primer name		Nucleotide sequence of primers and a probe (5’-3’)	Nucleotide position in HBV
HBV DNA, RNA-1816F	Forward	GCAACTTTTTCACCTCTGCCTA	1816-1837
HBV DNA, RNA-1974R	Reverse	GGAAAGAAGTCAGAAGGCAA	1974-1955
HBV DNA, RNA-1826F	Forward	CACCTCTGCCTAATCATC	1826-1843
HBV DNA, RNA-1947R	Reverse	AGTAACTCCACAGTAGCTCCAAATT	1947-1923
HBV DNA, RNA-Probe	Probe	(FAM)-TTCAAGCCTCCAAGCTGTGCCTTG-(TAMRA)	1863-1886

Nucleotide sequence of the primers and a probe

**Table:** 

Gene name		Nucleotide sequence of primers and a probe (5’-3’)
Transferrin receptor	Forward	GGACACCTATAAGGAACTGATTGAGA
	Reverse	AGTCCAGGTTCAATTCAACATCATG
	Probe	(FAM)-AATCACGAACTGACCAGCGACCTCTGC-(TAMRA)

### Quantitative gene expression analysis

Gene expression was analyzed with custom TaqMan Array plate and TaqMan Array 96-well plate, fast (Thermo Fisher Scientific) and the selected target genes were determined using the StepOne Plus real-time PCR system (Applied Biosystems) (Table 2). Expression levels of the target genes were analyzed using the ΔΔCt comparative threshold method. The GAPDH gene was used as an internal control.

**Table 2:** Primers used in this study. Primers used in custom TaqMan® Array Fast plate.

	Gene Symbol	Species	Dye	Assay ID	Company
1	18s rRNA	Human	FAM	Hs99999901_s1	Applied Biosystems
2	ABCC1	Human	FAM	Hs01561483_m1	Applied Biosystems
3	ABCC2	Human	FAM	Hs00960489_m1	Applied Biosystems
4	ABCG2	Human	FAM	Hs01053790_m1	Applied Biosystems
5	ACOX1	Human	FAM	Hs01074241_m1	Applied Biosystems
6	ACTB	Human	FAM	Hs99999903_m1	Applied Biosystems
7	ADSL	Human	FAM	Hs01075807_m1	Applied Biosystems
8	AHR	Human	FAM	Hs00169233_m1	Applied Biosystems
9	AKR1C1	Human	FAM	Hs04230636_sH	Applied Biosystems
10	APOBEC3A	Human	FAM	Hs02572821_s1	Applied Biosystems
11	APOBEC3B	Human	FAM	Hs00358981_m1	Applied Biosystems
12	APOBEC3G	Human	FAM	Hs00222415_m1	Applied Biosystems
13	ASL	Human	FAM	Hs00902699_m1	Applied Biosystems
14	ATG5	Human	FAM	Hs00355494_m1	Applied Biosystems
15	ATG7	Human	FAM	Hs00893766_m1	Applied Biosystems
16	BACH1	Human	FAM	Hs00230917_m1	Applied Biosystems
17	BCL2	Human	FAM	Hs04986394_s1	Applied Biosystems
18	BCL2L1	Human	FAM	Hs00236329_m1	Applied Biosystems
19	BDH1	Human	FAM	Hs00366297_m1	Applied Biosystems
20	BLVRA	Human	FAM	Hs00167599_m1	Applied Biosystems
21	BTRC	Human	FAM	Hs00182707_m1	Applied Biosystems
22	CASP1	Human	FAM	Hs00354836_m1	Applied Biosystems
23	CAT	Human	FAM	Hs00156308_m1	Applied Biosystems
24	CCL5	Human	FAM	Hs00982282_m1	Applied Biosystems
25	CHUK	Human	FAM	Hs00989502_m1	Applied Biosystems
26	CXCL9	Human	FAM	Hs00171065_m1	Applied Biosystems
27	FAH	Human	FAM	Hs00164611_m1	Applied Biosystems
28	FH	Human	FAM	Hs00264683_m1	Applied Biosystems
29	FTH1	Human	FAM	Hs01694011_s1	Applied Biosystems
30	G6PD	Human	FAM	Hs00166169_m1	Applied Biosystems
31	GAPDH	Human	FAM	Hs99999905_m1	Applied Biosystems
32	GCLC	Human	FAM	Hs00155249_m1	Applied Biosystems
33	GCLM	Human	FAM	Hs00978072_m1	Applied Biosystems
34	GPX1	Human	FAM	Hs00829989_gH	Applied Biosystems
35	GPX2	Human	FAM	Hs01591589_m1	Applied Biosystems
36	GSR	Human	FAM	Hs00167317_m1	Applied Biosystems
37	GSTA1	Human	FAM	Hs00275575_m1	Applied Biosystems
38	GSTM1	Human	FAM	Hs01683722_gH	Applied Biosystems
39	GSTP1	Human	FAM	Hs00943350_g1	Applied Biosystems
40	GSTZ1	Human	FAM	Hs01041668_m1	Applied Biosystems
41	HGD	Human	FAM	Hs01056732_m1	Applied Biosystems
42	HIF1A	Human	FAM	Hs00153153_m1	Applied Biosystems
43	HMOX1	Human	FAM	Hs01110250_m1	Applied Biosystems
44	HPD	Human	FAM	Hs00157976_m1	Applied Biosystems
45	IDH1	Human	FAM	Hs04966975_g1	Applied Biosystems
46	IFI16	Human	FAM	Hs00194261_m1	Applied Biosystems
47	IFI44L	Human	FAM	Hs00199115_m1	Applied Biosystems
48	IFNA1	Human	FAM	Hs00855471_g1	Applied Biosystems
49	IFNA2	Human	FAM	Hs00265051_s1	Applied Biosystems
50	IFNAR1	Human	FAM	Hs01066118_m1	Applied Biosystems
51	IFNG	Human	FAM	Hs00174143_m1	Applied Biosystems
52	IFNGR1	Human	FAM	Hs00166223_m1	Applied Biosystems
53	IKBKB	Human	FAM	Hs00233287_m1	Applied Biosystems
54	IKBKE	Human	FAM	Hs01063858_m1	Applied Biosystems
55	IL1B	Human	FAM	Hs01555410_m1	Applied Biosystems
56	IRF3	Human	FAM	Hs01547283_m1	Applied Biosystems
57	IRF7	Human	FAM	Hs01014809_g1	Applied Biosystems
58	IRF9	Human	FAM	Hs00196051_m1	Applied Biosystems
59	JAK1	Human	FAM	Hs01026983_m1	Applied Biosystems
60	JAK2	Human	FAM	Hs01078124_m1	Applied Biosystems
61	KEAP1	Human	FAM	Hs00202227_m1	Applied Biosystems
62	LIPH	Human	FAM	Hs00975890_m1	Applied Biosystems
63	MAF	Human	FAM	Hs04185012_s1	Applied Biosystems
64	ME1	Human	FAM	Hs00159110_m1	Applied Biosystems
65	ME2	Human	FAM	Hs00929809_g1	Applied Biosystems
66	NFKB1	Human	FAM	Hs00765730_m1	Applied Biosystems
67	NLRP3	Human	FAM	Hs00918082_m1	Applied Biosystems
68	NOTCH1	Human	FAM	Hs01062014_m1	Applied Biosystems
69	NQO1	Human	FAM	Hs01045993_g1	Applied Biosystems
70	OAS2	Human	FAM	Hs00942643_m1	Applied Biosystems
71	OSGIN1	Human	FAM	Hs00203539_m1	Applied Biosystems
72	PGD	Human	FAM	Hs00427230_m1	Applied Biosystems
73	PLA2G7	Human	FAM	Hs00965837_m1	Applied Biosystems
74	POMP	Human	FAM	Hs01106088_m1	Applied Biosystems
75	PRDX1	Human	FAM	Hs00602020_mH	Applied Biosystems
76	PSMA1	Human	FAM	Hs01027360_g1	Applied Biosystems
77	PSMB5	Human	FAM	Hs00605652_m1	Applied Biosystems
78	RAD51	Human	FAM	Hs00947967_m1	Applied Biosystems
79	RELA	Human	FAM	Hs00153294_m1	Applied Biosystems
80	RXRA	Human	FAM	Hs01067640_m1	Applied Biosystems
81	SDHA	Human	FAM	Hs07291714_mH	Applied Biosystems
82	SETD2	Human	FAM	Hs00383442_m1	Applied Biosystems
83	SIRT1	Human	FAM	Hs01009006_m1	Applied Biosystems
84	SLC7A11	Human	FAM	Hs00921938_m1	Applied Biosystems
85	SOD1	Human	FAM	Hs00533490_m1	Applied Biosystems
86	SOD2	Human	FAM	Hs00167309_m1	Applied Biosystems
87	SQSTM1	Human	FAM	Hs01061917_g1	Applied Biosystems
88	SRXN1	Human	FAM	Hs00607800_m1	Applied Biosystems
89	STAT1	Human	FAM	Hs01013996_m1	Applied Biosystems
90	STAT2	Human	FAM	Hs01013123_m1	Applied Biosystems
91	SYVN1	Human	FAM	Hs00381211_m1	Applied Biosystems
92	TAT	Human	FAM	Hs00356930_m1	Applied Biosystems
93	TNF	Human	FAM	Hs01113624_g1	Applied Biosystems
94	TXN	Human	FAM	Hs01555214_g1	Applied Biosystems
95	TXNRD1	Human	FAM	Hs00917067_m1	Applied Biosystems
96	ULK1	Human	FAM	Hs00177504_m1	Applied Biosystems

Primers used in this study

**Table:** 

	Gene Symbol	Species	Dye	Assay ID	Company
	STAT1	Human	FAM	Hs01013996_m1	Applied Biosystems
	IRF2	Human	FAM	Hs01082884_m1	Applied Biosystems
	IFI44L	Human	FAM	Hs00915292_m1	Applied Biosystems
	FAH	Human	FAM	Hs00164611_m1	Applied Biosystems
	NNMT	Human	FAM	Hs00196287_m1	Applied Biosystems
	NFE2L2	Human	FAM	Hs00975961_g1	Applied Biosystems

### Microarray analysis

Microarray analyses were performed by Takara Bio using the SurePrint G3 Human GE 8 ×  60 K v3 Microarray (Agilent Technologies), as described previously [11, 12]. The microarray data were validated and normalized using GeneSpring ver. 14.9.1 software (Agilent Technologies) by Hokkaido System Science Co., Ltd. (Hokkaido, Japan). The median shift normalization to 75 percentile and baseline transformation using the median of the control samples were applied. Probes with 100.0% of samples in any one of two conditions were flagged as [Detected] and gene expression values less than the cut-off value (10.0) were excluded to remove spots with low signal values and low reliability. The raw microarray data were deposited in the NCBI Gene Expression Omnibus (GEO) under the accession number GSE253496 (https://www.ncbi.nlm.nih.gov/geo/query/acc.cgi?acc=GSE253496). Functional annotation was performed on the samples using the gene set enrichment analysis (GSEA) method [15], supported by the Broad Institute website (https://www.gsea-msigdb.org/gsea/index.jsp) and performed using GSEA software (version 4.3.2; Broad Institute, Inc., Massachusetts Institute of Technology, Boston, MA, USA, and Regents of the University of California, CA, USA). False-discovery rate (FDR) q-values <  0.25 and adjusted p values <  0.05 were considered significant enrichment.

### Small interfering RNA

siRNA constructs were obtained using siGENOME SMARTpool reagents (Horizon Discovery, Lafayette, CO, USA) that targeted siGENOME SMARTpool and siGENOME Non-Targeting Control siRNA (Table 3) [12]. We used an siGENOME SMARTpool Cherry-pick library (Horizon Discovery) of 43 candidate genes to screen for RNA interference (Table 3). PXB cells and HepG2. D11 cells were transfected with 25 nM siRNA using DharmaFECT 4 transfection reagent (Horizon Discovery).

**Table 3:** Catalog number and descriptions lists of siRNA.

Pool Catalog Number	Gene Symbol	Gene Accession	Description
M-003543-01	STAT1	6772	STAT1 siGENOME SMART - Human
M-011705-01	IRF2	3660	IRF2 siGENOME SMART - Human
M-004599-00	IFI44L	10964	IFI44L siGENOME SMART - Human
D-001206-13			siGENOME Non-Targeting Control siRNA Pool #1

Cherry-Pick Custom Library (siRNA)

**Table:** 

	Pool Catalog Number	Gene Symbol	Gene Accession	Description
1	M-009821-00	DPYSL3	1809	DPYSL3 siGENOME SMARTpool - Human
2	M-012559-02	IGFBP1	3484	IGFBP1 siGENOME SMARTpool - Human
3	M-009635-00	FAH	2184	FAH siGENOME SMARTpool - Human
4	M-008336-00	TAT	6898	TAT siGENOME SMARTpool - Human
5	M-010855-01	DDIT4	54541	DDIT4 siGENOME SMARTpool - Human
6	M-014727-00	RTP3	83597	RTP3 siGENOME SMARTpool - Human
7	M-008506-00	TDO2	6999	TDO2 siGENOME SMARTpool - Human
8	M-009910-01	HSD11B1	3290	HSD11B1 siGENOME SMARTpool - Human
9	M-009946-01	GPAM	57678	GPAM siGENOME SMARTpool - Human
10	M-003410-01	NR0B2	8431	NR0B2 siGENOME SMARTpool - Human
11	M-008286-01	CYP2C8	1558	CYP2C8 siGENOME SMARTpool - Human
12	M-008822-01	ATF5	22809	ATF5 siGENOME SMARTpool - Human
13	M-003746-02	MAF	4094	MAF siGENOME SMARTpool - Human
14	M-004819-03	DDIT3	1649	DDIT3 siGENOME SMARTpool - Human
15	M-003754-01	TRIB3	57761	TRIB3 siGENOME SMARTpool - Human
16	M-008169-00	CYP3A4	1576	CYP3A4 siGENOME SMARTpool - Human
17	M-008726-00	AQP7	364	AQP7 siGENOME SMARTpool - Human
18	M-004297-01	SLC7A2	6542	SLC7A2 siGENOME SMARTpool - Human
19	M-010351-01	NNMT	4837	NNMT siGENOME SMARTpool - Human
20	M-007966-02	IL1RN	3557	IL1RN siGENOME SMARTpool - Human
21	M-007443-01	SLC22A1	6580	SLC22A1 siGENOME SMARTpool - Human
22	M-003964-01	DUSP6	1848	DUSP6 siGENOME SMARTpool - Human
23	M-004777-01	IGFBP3	3486	IGFBP3 siGENOME SMARTpool - Human
24	M-009703-00	OGDHL	55753	OGDHL siGENOME SMARTpool - Human
25	M-022006-01	EXOC3L4	91828	EXOC3L4 siGENOME SMARTpool - Human
26	M-017077-00	PPP1R3C	5507	PPP1R3C siGENOME SMARTpool - Human
27	M-021435-00	INHBE	83729	INHBE siGENOME SMARTpool - Human
28	M-015771-00	G0S2	50486	G0S2 siGENOME SMARTpool - Human
29	M-018337-01	MT1X	4501	MT1X siGENOME SMARTpool - Human
30	M-003265-01	FOS	2353	FOS siGENOME SMARTpool - Human
31	M-003723-02	IKBKE	9641	IKBKE siGENOME SMARTpool - Human
32	M-011179-00	GSTP1	2950	GSTP1 siGENOME SMARTpool - Human
33	M-010723-02	S100A14	57402	S100A14 siGENOME SMARTpool - Human
34	M-027199-01	SALL2	6297	SALL2 siGENOME SMARTpool - Human
35	M-020868-01	DCDC2	51473	DCDC2 siGENOME SMARTpool - Human
36	M-013833-03	ANGPTL8	55908	ANGPTL8 siGENOME SMARTpool - Human
37	M-009324-01	TBXAS1	6916	TBXAS1 siGENOME SMARTpool - Human
38	M-004529-02	NCF2	4688	NCF2 siGENOME SMARTpool - Human
39	M-005472-02	CXCR3	2833	CXCR3 siGENOME SMARTpool - Human
40	M-003543-01	STAT1	6772	STAT1 siGENOME SMARTpool - Human
41	M-010995-01	APOA4	337	APOA4 siGENOME SMARTpool - Human
42	M-015096-01	BEX1	55859	BEX1 siGENOME SMARTpool - Human
43	M-025114-01	HMGCLL1	54511	HMGCLL1 siGENOME SMARTpool - Human
44	D-001206-13			siGENOME Non-Targeting Control siRNA Pool #1

### Vector transfection

The cytomegalovirus vector used to overexpress STAT1 [pRP[Exp]-CMV > hSTAT1 (NM_001384891.1)] and FAH [pRP[Exp]-CMV > hFAH (NM_001374380.1)] was constructed and packaged using VectorBuilder. Further information is available on the VectorBuilder website (https://en.vectorbuilder.com/) under the VectorBuilder ID, STAT1: VB230117-1096kjt, FAH: VB230117-1091yve. The plasmid vector was transfected into HepG2.D11 cells using Lipofectamine LTX Reagent (Thermo Fisher Scientific).

### Fumarate assay

The abundance of intracellular fumarate was assessed using a fumarate colorimetric assay kit (Sigma-Aldrich, MAK060) that uses an enzyme assay. Briefly, cells (1 × 106) were collected and homogenized in 100 µ L of Fumarate Assay Buffer and centrifuged the sample at 13,000 × g for 10 min to remove insoluble material. Absorbance was measured at 450 nm and compared to standard curves, according to the manufacturer’s instructions.

### MTT assay

Cell viability was determined using the Cell Counting kit-8 (Dojindo, Kumamoto, Japan), a modified 3-(4,5-di-methylthiazol-2-yl)-2,5-diphenyltetrazolium bromide (MTT) assay. The optical density values of untreated and vehicle-treated cells were compared to obtain the ratios of cell numbers.

### Western blotting

HepG2.D11 cells were prepared using radioimmunoprecipitation assay (RIPA) lysis buffer (50 mmol/L Tris-HCl buffer [pH7.6], 150 mmol/L NaCl, 1% Nonidet^®^ P40 Substitute, 0.5% Sodium Deoxycholate, 0.1% SDS) (Nakarai Tesuque, Kyoto, Japan). Anti-STAT1 antibody (Cell Signaling Technology, Danvers, MA, USA, #9175), anti-FAH (Sigma-Aldrich, HPA041370) antibody, and anti-β-actin (D6A8) monoclonal antibody (Cell Signaling Technology) were used for protein detection. Immune complexes were visualized using enhanced chemiluminescence detection reagents (Amersham Biosciences, Piscataway, NJ, USA) according to the manufacturer’s protocol.

### Statistical analyses

Statistical analyses were performed using GraphPad Prism software (version 10; GraphPad Software Inc., San Diego, CA, USA). Statistical significance was determined using Mann-Whitney U tests or one-way analysis of variance followed by the Tukey–Kramer post-hoc test [12]. p values <  0.05 were considered statistically significant. Any comparisons not shown on graphs are non-significant.

## Results

### Knockdown of STAT1 increased viral products in HBV-transfected HepG2 cells, but decreased in the primary human hepatocyte infection system

**Fig 1:**
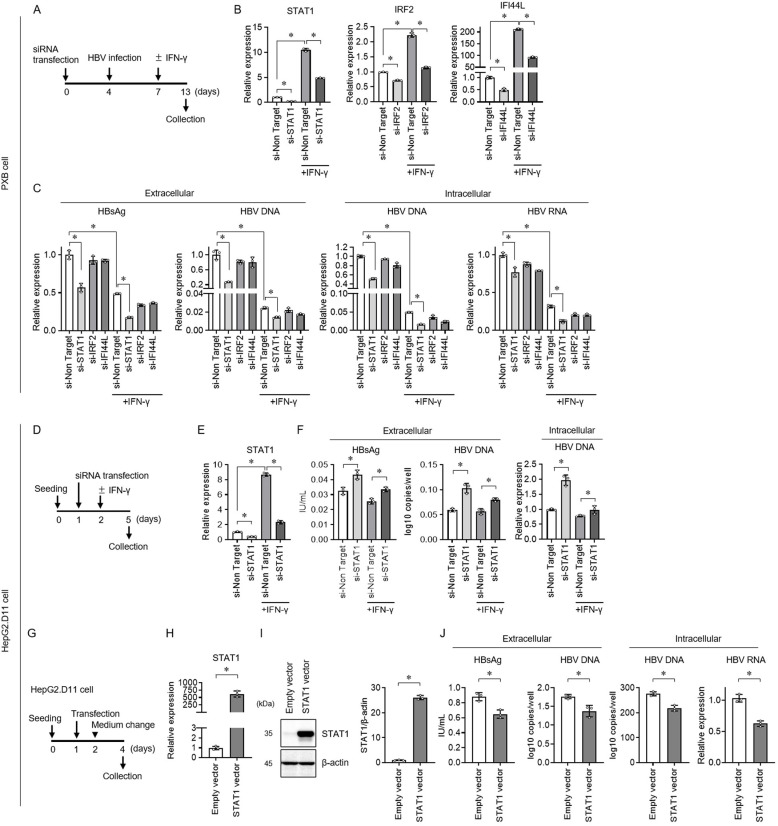
Host factor gene knockdown analysis in primary human hepatocyte hepatitis B virus (HBV) infection system and HBV-transfected HepG2 cells. (A) siRNA was transfected into the PXB cells on day 0 and HBV was added on day 4. IFN-γ was diluted and added to the medium on day 7 and the supernatant and PXB cells were collected on day 13. In the control group, only medium was added. The culture medium was exchanged on day 4, 6, and 7. (B) mRNA expression of STAT1, IRF2, and IFI44L, and (C) extracellular hepatitis B surface antigen (HBsAg) and HBV DNA and intracellular HBV DNA and HBV RNA were analyzed on day 13. (D) HepG2.D11 cells were seeded on day 0 and siRNA was transfected at medium change on day 1. IFN-γ was diluted and added to the medium on day 2 and the supernatant and HepG2.D11 cells were collected on day 5. In the control group, only medium was added. (E) mRNA expression of STAT1 and (F) extracellular HBsAg and HBV DNA and intracellular HBV DNA was analyzed on day 5. (G) HepG2.D11 cells were seeded on day 0 and CMV vector inserted cDNA STAT1 was transfected on day 1. The culture medium was exchanged on day 2 and the supernatant and HepG2.D11 cells were collected on day 4. (H) mRNA expression of STAT1 and (I) western blot analysis of extracts from HepG2.D11 using STAT1 antibody and normalized to β-actin on day 4. (J) Extracellular HBsAg and HBV DNA and intracellular HBV DNA and HBV RNA were analyzed on day 4. Data are presented as the mean ±  standard deviation (n =  3). (D-F) and (G-J) The experiments were replicated three times. * p <  0.05 using the Mann-Whitney U test. Abbreviations: HBsAg, hepatitis B surface antigen; HBV, hepatitis B virus; IFN, interferon; IRF, interferon regulatory factor; PXB, primary human hepatocyte; siRNA, small interfering RNA; STAT1, signal transducer and activator of transcription 1.

siRNA transfection experiments were performed using an *in vitro* HBV infection assay system with PXB cells, together with the reported host immune-related genes, IRF2 [16] and IFI44L [12] (Fig 1A). IFN-γ significantly increased the expression of STAT1, IRF2, and IFI44L in PXB cells (Fig 1B). siRNA transfection decreased the expression of each target gene in the presence/absence of IFN-γ. Compared with IRF2 and IFI44L knockdown, STAT1 knockdown significantly reduced extracellular HBsAg and HBV DNA and intracellular HBV DNA and HBV RNA levels (Fig 1C). IFN-γ treatment significantly reduced HBV transfection levels, and knockdown of STAT1 further reduced HBV replication levels in the presence of IFN-γ. Next, we performed siRNA transfection experiments targeting the STAT1 gene using HepG2 cells transfected with HBV, exhibiting stable production of HBsAg and HBV in the supernatant (HepG2.D11 cells) (Fig 1D). Similar to PXB cells, IFN-γ significantly increased STAT1 gene expression in HepG2.D11 cells, and siRNA transfection decreased STAT1 expression (Fig 1E). In contrast to PXB cells, STAT1 knockdown in HepG2.D11 cells resulted in increased extracellular HBsAg and HBV DNA levels and intracellular of HBV DNA levels (Fig 1F). To overexpress STAT1 in HepG2.D11 cells, STAT1 vector was transfected (Fig 1G). Increased STAT1 expression was confirmed by quantitative real-time polymerase chain reaction (PCR) (Fig 1H) and western blot (Fig 1I). Overexpression of STAT1 decreased extracellular HBsAg and HBV DNA and intracellular HBV DNA and RNA levels (Fig 1J). These results indicate that the host gene STAT1 showed anti-HBV activity in conventional HBV-transfected HepG2 cells, whereas in the primary human hepatocyte HBV infection system, STAT1 knockdown conversely reduced HBV replication capacity.

### RNA microarray analysis in knockdown of STAT1 in primary human hepatocyte HBV infection system and HBV-transfected HepG2 cells

**Fig 2:**
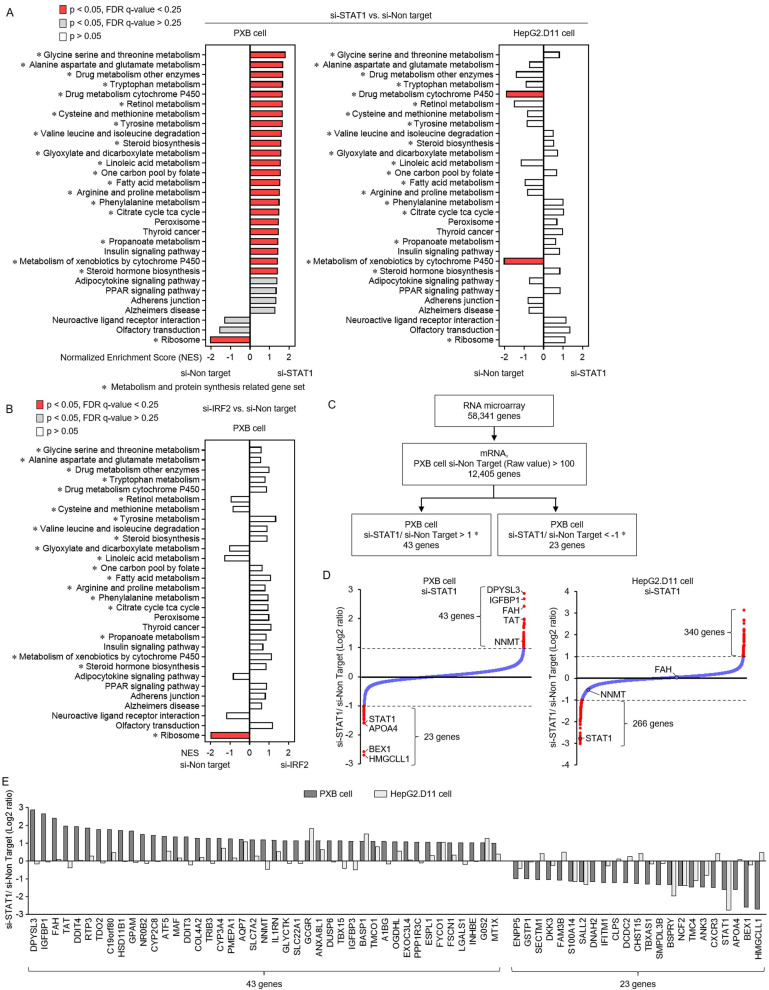
RNA microarray analysis in knockdown of STAT1 in primary human hepatocyte HBV infection system and HBV-transfected HepG2 cells. (A–E) RNA microarray was performed in HBV-infected PXB cells and HepG2.D11 cells with STAT1 knockdown and HBV-infected PXB cells with IRF2 knockdown. siRNA was transfected into the PXB cells on day 0 and HBV was added on day 4 and PXB cells were collected on day 13. HepG2.D11 cells were seeded on day 0 and siRNA was transfected at medium change on day 1. HepG2.D11 cells were collected on day 5. (A, B) Results of the Gene Set Enrichment Analysis (GSEA) of the Kyoto Encyclopedia of Genes and Genomes (KEGG) pathway. Bars in red indicate significant enrichment at FDR <  0.25, bars in gray represent gene sets with FDR >  0.25 and a nominal p value <  0.05 and bars in white represent gene sets with a nominal p value >  0.05. A positive normalized enrichment score (NES) value indicates enrichment in the si-STAT1 in PXB cells (left) and HepG2.D11 cells (right) (A) and si-IRF2 in PXB cells (B). *  The gene sets related metabolism and protein synthesis. (C) The algorithm to extract genes changed by STAT1 knockdown in HBV-infected PXB cells. (D) Relative changes [si-STAT1/ si-Non-Target (Log2 ratio)] of 12,405 genes [PXB cell si-Non-Target (Raw value) >  100] in STAT1 knockdown in HBV-infected PXB cells and HepG2.D11 cells. (E) Relative changes of 63 genes (43 up- and 23 down-regulated genes) extracted by the algorithm in STAT1 knockdown of PXB cells and HepG2.D11 cells. Abbreviations: FDR, false discovery rate; GSEA, Gene Stet Enrichment Analysis; HBsAg, hepatitis B surface antigen; HBV, hepatitis B virus; IFN, interferon; IRF, interferon regulatory factor; KEGG, Kyoto Encyclopedia of Genes and Genomes; PXB, primary human hepatocyte; siRNA, small interfering RNA; STAT1, signal transducer and activator of transcription 1.

To investigate the mechanism by which STAT1 knockdown reduces HBV levels in primary human hepatocytes, RNA microarray analysis was performed to comprehensively examine the molecular changes in PXB cells. In HBV-infected PXB cells, STAT1 knockdown enriched 24 gene sets (p <  0.05, FDR q-value <  0.25) (Fig 2A). Of the 24 gene sets, 21 were associated with metabolism and protein synthesis. However, STAT1 knockdown in HepG2.D11 cells and IRF2 knockdown in PXB cells did not result in similar molecular changes (Fig 2B). From the 58,341 genes, 12,405 mRNAs were extracted after excluding genes with low signal values and low reliability [PXB cell si-Non-Target (Raw value) >  100] (Fig 2C). In HBV-infected PXB cells, STAT knockdown increased expression of 43 genes [si-STAT1/si-Non-Target (Log2 ratio) >  1] and decreased expression of 23 genes [si-STAT1/si-Non-Target (Log2 ratio) < -1] (Figs 2C–E). In HepG2.D11 cells, STAT knockdown increased expression of 340 genes [si-STAT1/si-Non-Target (Log2 ratio) >  1] and decreased expression of 266 genes [si-STAT1/si-Non-Target (Log2 ratio) < -1] (Fig 2D).

### A comprehensive functional screen identified FAH as a candidate host factor that regulates HBV infection

**Fig 3:**
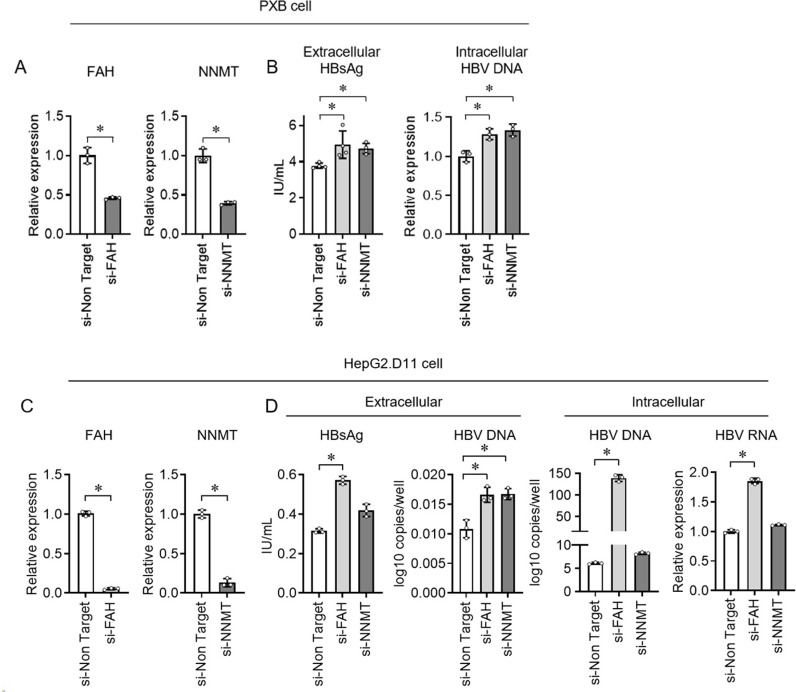
Functional screen analysis of two candidate genes in HBV infection assay systems. (A, B) siRNA of FAH and NNMT were transfected into the PXB cells on day 0 and HBV was added on day 4. Supernatant and PXB cells were collected on day 13. The culture medium was exchanged on day 4, 6, and 7. (A) mRNA expression of FAH and NNMT, and (B) extracellular hepatitis B surface antigen (HBsAg) and intracellular HBV DNA were analyzed on day 13. (C, D) HepG2.D11 cells were seeded on day 0 and siRNA of FAH and NNMT was transfected at medium change on day 1. HepG2.D11 cells were collected on day 5. (C) mRNA expression of FAH and NNMT and (D) extracellular HBsAg and HBV DNA and intracellular HBV DNA and HBV RNA were analyzed on day 5. (A–D) Data are represented as mean ±  standard deviation (n =  3). (A, B) and (C, D) The experiments were replicated three times. (A, C) Mann-Whitney U test. (B, D) Tukey–Kramer post-hoc test. * p <  0.05. Abbreviations: FAH, fumarylacetoacetate hydrolase; HBsAg, hepatitis B surface antigen; HBV, hepatitis B virus; NNMT, nicotinamide N-methyltransferase; PXB, primary human hepatocyte; siRNA, small interfering RNA; STAT1, signal transducer and activator of transcription 1.

As STAT1 knockdown in HBV-infected PXB cells suppressed HBV replication levels, we investigated genes with anti-HBV activity among the candidate molecules. Forty-three genes that were highly expressed in PXB cells were identified using microarray analysis. siRNA transfection of the 43 genes was performed using HBV-infected PXB cells. siRNA transfection of 12 genes increased extracellular HBsAg levels by more than 1.2-fold (S1 Fig A). To identify candidate genes with a STAT1-independent anti-HBV effect, double siRNA transfection with STAT1 was performed on each of these 12 genes (S1 Fig B). With STAT1 knockdown, two genes, FAH and nicotinamide N-methyltransferase (NNMT), showed a more than 1.5-fold increase in HBsAg levels. In the STAT1 knockdown condition, knockdown of FAH significantly elevated intracellular HBV DNA. The knockdown efficiency was confirmed in two genes, FAH and NNMT (Fig 3A). Knockdown of FAH and NNMT significantly elevated extracellular HBsAg and intracellular HBV DNA levels (Fig 3B). FAH and NNMT siRNA transfection experiments were performed in HepG2.D11 cells (Figs 3C and D). Knockdown of FAH significantly elevated extracellular HBsAg, HBV DNA, intracellular HBV DNA, and HBV RNA levels and increased HBV replication levels compared to NNMT. These results suggest that FAH has anti-HBV effects, as determined by siRNA screening of genes altered by STAT1 knockdown in PXB cells. FAH knockdown resulted in increased HBV replication in the primary human hepatocyte infection system and conventional HBV-transfected HepG2 clone.

### Elevated FAH gene expression inhibits HBV replication

**Fig 4:**
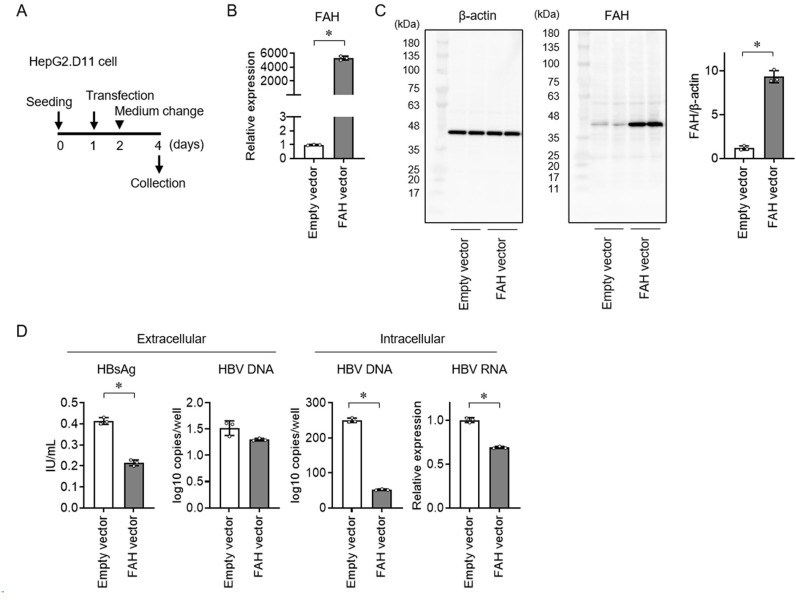
Host gene FAH vector transfection in HBV-transfected HepG2 cells. (A) HepG2.D11 cells were seeded on day 0 and CMV vector inserted cDNA FAH was transfected on day 1. The culture medium was exchanged on day 2 and the supernatant and HepG2.D11 cells were collected on day 4. (B) mRNA expression of FAH and (C) western blot analysis of extracts from HepG2.D11 using FAH antibody and normalized to β-actin on day 4. (D) Extracellular HBsAg and HBV DNA and intracellular HBV DNA and HBV RNA were analyzed on day 4. Data are represented as mean ±  standard deviation (n =  3). The experiments were replicated three times. * p <  0.05 using the Mann-Whitney U test. Abbreviations: CMV, cytomegalovirus; FAH, fumarylacetoacetate hydrolase; HBsAg, hepatitis B surface antigen; HBV, hepatitis B virus.

To overexpress FAH in HepG2.D11 cells, FAH cDNA was inserted into a plasmid vector and transfected (Fig 4A). Increased FAH expression was confirmed using qRT-pCR and western blot (Figs 4B and C). Overexpression of FAH resulted in decreased extracellular HBsAg and intracellular HBV DNA and RNA levels (Fig 4D). These results suggest that elevated FAH expression in hepatocytes inhibits HBV replication.

### Dimethyl fumarate exhibited antiviral effects by inducing autophagy and anti-HBV-related genes

**Fig 5:**
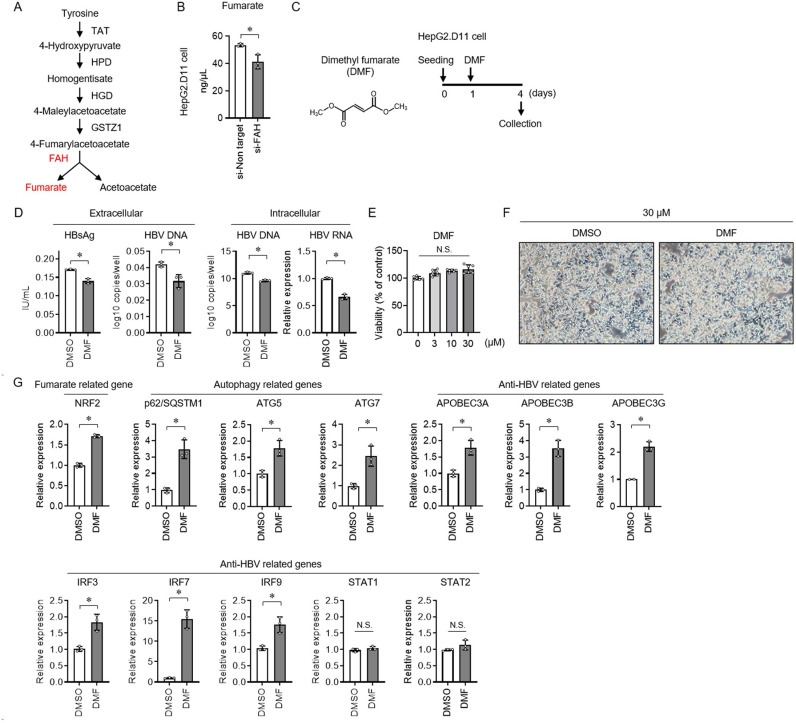
Anti-HBV effect of dimethyl fumarate and gene expression analysis in hepatocytes. (A) Diagram of the tyrosine metabolic pathway. The converting 4-fumarylacetoacetate to fumarate and acetoacetate is catalyzed by FAH. (B) HepG2.D11 cells were transfected with siRNA of FAH, and cells were collected 72 h later and intracellular fumaric acid concentration was measured. (C) Molecular structure of DMF (left). HepG2.D11 cells were seeded on day 0, DMF was added to the culture supernatant on day 1; supernatant and HepG2.D11 cells were collected on day 4 (right). (D) DMF and DMSO were added to HepG2.D11 cells at 30 μM and analyzed for extracellular HBsAg and HBV DNA and intracellular HBV DNA and HBV RNA on day 4. (E) DMF and DMSO were added to HepG2.D11 cells at 0, 3, 10, and 30 µ M, and MTT assay was performed after 72 h to calculate cell viability. (F, G) DMF and DMSO were added to HepG2.D11 cells at 30 μM on day 1 and captured by phase contrast microscopy on day 4. Images were obtained from a ×4 objective (scale bar, 200 μm) (F). HepG2.D11 cells were collected on day 4 and mRNA expression was analyzed by real-time quantitative reverse transcription polymerase chain reaction (G). Data are represented as mean ±  standard deviation (n =  3). (C-F) The experiments were replicated three times. * p <  0.05 using the Mann-Whitney U test.

FAH is the terminal step in the tyrosine catabolic pathway. The conversion of 4-fumarylacetoacetate to fumarate and acetoacetate is catalyzed by FAH [17] (Fig 5A). FAH knockdown in HepG2.D11 cells reduced intracellular fumarate levels (Fig 5B). Dimethyl fumarate (DMF), the methyl ester of fumaric acid, is a cell-permeable fumarate derivative [18] (Fig 5C). In HepG2.D11 cells, DMF decreased extracellular HBsAg and HBV DNA levels and intracellular HBV DNA and RNA levels (Fig 5D). No cytotoxicity was seen in DMF at concentrations of 0–30 μM (Fig 5E), and no difference in the morphology of cells was observed by microscopic examination (Fig 5F). To examine the association between DMF and the antiviral response, the expression of intracellular molecules was analyzed using qRT-PCR (Fig 5G and Table 2). Referring to papers related to DMF and antiviral functions, 96 molecules were selected (including internal controls) [11,12,19–22]. DMF increased nuclear erythroid-related factor 2 (NRF2) in HepG2.D11 cells. Autophagy-related molecules, such as p62/sequestosome 1 (SQSTM1), autophagy-related gene (ATG)5, and ATG7, were upregulated by DMF. DMF induced antiviral molecules, including apolipoprotein B mRNA editing enzyme catalytic subunit (APOBEC)3A, APOBEC3B, and APOBEC3G, and interferon regulatory factor (IRF)3, IRF5, and IRF7. The anti-HBV molecules STAT1 and STAT2 were not induced by DMF. These results suggest that FAH promotes fumarate production and induces NRF2 and autophagy- and anti-HBV-related genes to exhibit antiviral effects in a STAT1/2-independent manner.

## Discussion

In the STAT1 knockdown experiment, the viral products were elevated in the conventional HBV-transfected HepG2 cells but decreased in the primary human hepatocyte infection system. RNA microarray analysis showed that STAT1 knockdown induced changes in intracellular molecules related to metabolism and protein synthesis in PXB cells, but not in HepG2.D11 cells. Comprehensive functional screening identified FAH as a candidate host factor that controls HBV infection independent of STAT1. DMF, the methyl ester of the FAH metabolite, showed antiviral effects and the expression of genes related to autophagy and anti-HBV effects were altered.

With the downregulation of STAT1, HBV gene expression was suppressed in primary human hepatocytes in an *in vitro* HBV infection assay system, whereas expression was enhanced in HepG2 cells transfected with the viral genome. Wilkening *et al.* performed a comparison of primary hepatocytes and hepatoma cell line HepG2 in the presence of different classes of promutagens [23]. The three promutagens caused DNA damage in primary human hepatocytes, but not in HepG2 cells. The most abundant isozyme of all P450s in the human liver, CYP3A4, is the most important isoform in drug metabolism in primary hepatocytes; however, CYP3A4 mRNA was not detected in HepG2 cells. In addition, the researchers detected mRNA P450 in primary hepatocytes, similar to that previously reported for human liver samples [24, 25]. Similarly, in this study, host gene expression in primary hepatocytes of the *in vitro* HBV infection assay system was markedly different from that in HepG2 cells with downregulation of STAT1.

A comprehensive functional screen of genes altered by STAT1 knockdown in PXB cells identified FAH as the gene exhibiting anti-HBV activity. FAH is the last enzyme in the tyrosine catabolic pathway [26] and catalyzes the breakdown of fumarylacetoacetate into fumarate and acetoacetate [27].

In this study, knockdown of FAH in HepG2.D11 cells resulted in a marked (25-fold) increase in intracellular HBV DNA levels. In contrast, the increase in intracellular HBV RNA levels was about 2-fold. In HBV life cycle, the pregenomic RNA (pgRNA) is packaged with polymerase protein into immature nucleocapsids which consist of a core protein and are then reverse-transcribed into relaxed circular DNA (rcDNA) [28]. The variation in intracellular DNA and RNA levels upon FAH knockdown might indicate the possibility that FAH affects the pgRNA incorporation into the nucleocapsid and/or its reverse transcription process during the HBV life cycle. Moreover, overexpression or knockdown of FAH in HepG2.D11 cells resulted in less pronounced changes in extracellular HBV DNA than in intracellular HBV DNA and HBV RNA or extracellular HBsAg. This is an important point for further functional evaluation of the FAH molecules extracted in this screening experiment, and we plan to clarify this point in a future experiment.

DMF, a fumarate derivative, is used as an immunomodulatory drug, especially in the treatment of multiple sclerosis [29]. In alcohol-related liver disease, DMF suppresses the inflammatory response and ameliorates hepatitis and lipidosis [30]. DMF may also be involved in cellular immune responses and antiviral defense mechanisms [31, 32].

DMF enhances the activity of NRF2, a transcription factor that regulates the expression of various antioxidant proteins and detoxification enzymes [33, 34]. NRF2 modulates p62/SQSTM1 which is a protein that targets ubiquitinated proteins for autophagic degradation, as well as autophagy initiating proteins such as ATG5/7 [19,35,36]. Autophagy plays an important role in HBV-related innate and adaptive immune responses [37]. Miyakawa *et al.* reported that galectin-9 suppresses HBV replication by selective autophagy of the viral core protein via p62/SQSTM1 [35,38]. In this study, DMF exhibited antiviral effects and increased NRF2 and autophagy-related gene expression. As shown in these papers, it is possible that autophagy may be involved in the antiviral effects of this study.

In this study, DMF also induced the expression of antiviral molecules such as APOBEC3A/B/G and IRF3/5/7 in a STAT1/2-independent manner. The APOBEC protein family, including APOBEC3A/B/G interferes with cccDNA stability through cytidine deamination and apurinic/apyrimidinic site formation [39–41]. The IRF family of transcription factors plays a critical role in the human innate immune response, with IFN production being a hallmark consequence of activation [42, 43]. These reports suggest that in this study, increased gene expression was associated with the anti-HBV effect of DMF. However, the exact gene signaling pathways and mechanisms in hepatocytes are unknown, and this is a great interest as the next subject.

In this study, we were focused on the influence of host factors on HBV transcription and amplification in hepatocytes. Transfection experiments in HepG2 cells were performed to examine intracellular viral transcription and amplification. We consider that there is no difference between HepG2 and HepG2-NTCP cells with respect to intracellular viral transcription and amplification. Using PXB cells, it is possible to observe viral infection. We performed experiments in which the order of HBV infection and siRNA knockdown was switched in the molecules we have examined, including STAT1. Gene knockdown did not affect the process of HBV entry into hepatocytes (S2 Fig). HBV infection experiments using HepG2-NTCP cells, which are not commercially available, are being considered as the next project.

In conclusion, knockdown of the host gene STAT1 showed antiviral activity in conventional HBV-transfected HepG2 cells, but conversely decreased viral replication in a primary human hepatocyte infection system. STAT1 knockdown induced changes in intracellular molecules related to metabolism and protein synthesis in PXB cells, but not in HepG2.D11 cells. FAH was identified as a candidate host factor that contributes to the control of viral infection independent of STAT1, and its metabolite, DMF, exhibited antiviral activity. These results suggest that the novel host factor, FAH, and its metabolites may be an innovative therapeutic strategy for controlling the HBV life cycle.

## Supporting information

S1 Fig Functional screen analysis of two candidate genes in HBV infection assay systems. (A) siRNA of 43 candidate genes from the Cherry-pick library was transfected into the PXB cells on day 0 and HBV was added on day 4. The extracellular hepatitis B surface antigen (HBsAg) was analyzed on day 13. The culture medium was exchanged on days 4, 6, and 7. The 1.2-fold value of HBsAg in the sample transfected with the si-Non-Target is indicated by the dotted line. (B) Double siRNA of 12 target genes and STAT1 was transfected into the PXB cells on day 0 and HBV was added on day 4. The extracellular HBsAg and intracellular HBV DNA was analyzed on day 13. The 1.5-fold value of HBsAg in samples transfected with si-STAT1 is indicated by the dotted line. Abbreviations: HBsAg, hepatitis B surface antigen; HBV, hepatitis B virus; PXB, primary human hepatocyte; siRNA, small interfering RNA.
(TIF)

S2 Fig An experiment in which the order of HBV infection and siRNA knockdown was reversed in PXB cells. (A) HBV was added on day 0 and siRNA was transfected into the PXB cells on day 2. The supernatant and PXB cells were collected on day 13. The culture medium was exchanged on day 2, 4, and 7. (B) mRNA expression of STAT1 and (C) extracellular HBsAg and HBV DNA and intracellular HBV DNA and HBV RNA were analyzed on day 13.
(TIF)

S3 Fig (TIF)

## Abbreviations

APOBEC: apolipoprotein B mRNA editing enzyme catalytic subunit
ATG: autophagy-related gene
cccDNA: covalently closed circular DNA
CHB: chronic hepatitis B
DMF: dimethyl fumarate
FAH: fumarylacetoacetate hydrolase
HBsAg: hepatitis B surface antigen
HBV: hepatitis B virus
HCC: hepatocellular carcinoma
IFN: interferon
IRF: interferon regulatory factor
JAK: Janus kinase
MOI: multiplicity of infection
MTT: 3-(4,5-di-methylthiazol-2-yl)-2,5-diphenyltetrazolium bromide
NES: normalized enrichment score
NNMT: nicotinamide N-methyltransferase
NRF: nuclear erythroid-related factor
NUC: nucleotide/nucleoside analog
PEG-IFN-γ: pegylated interferon-γ
PXB: primary human hepatocyte
qRT-PCR: quantitative real-time polymerase chain reaction
SCID: severe combined immunodeficiency
siRNA: small interfering RNA
SQSTM1: sequestosome 1
STAT: signal transducer and activator of transcription
uPA: urokinase-type plasminogen activator.
