# Supplementary figures and images for: Identification of novel antiviral host factors by functional gene expression analysis using in vitro HBV infection assay systems
Source: PLoS One. 2025 Mar 6;20(3):e0314581. doi: 10.1371/journal.pone.0314581 (PMC11884705; doi:10.1371/journal.pone.0314581)

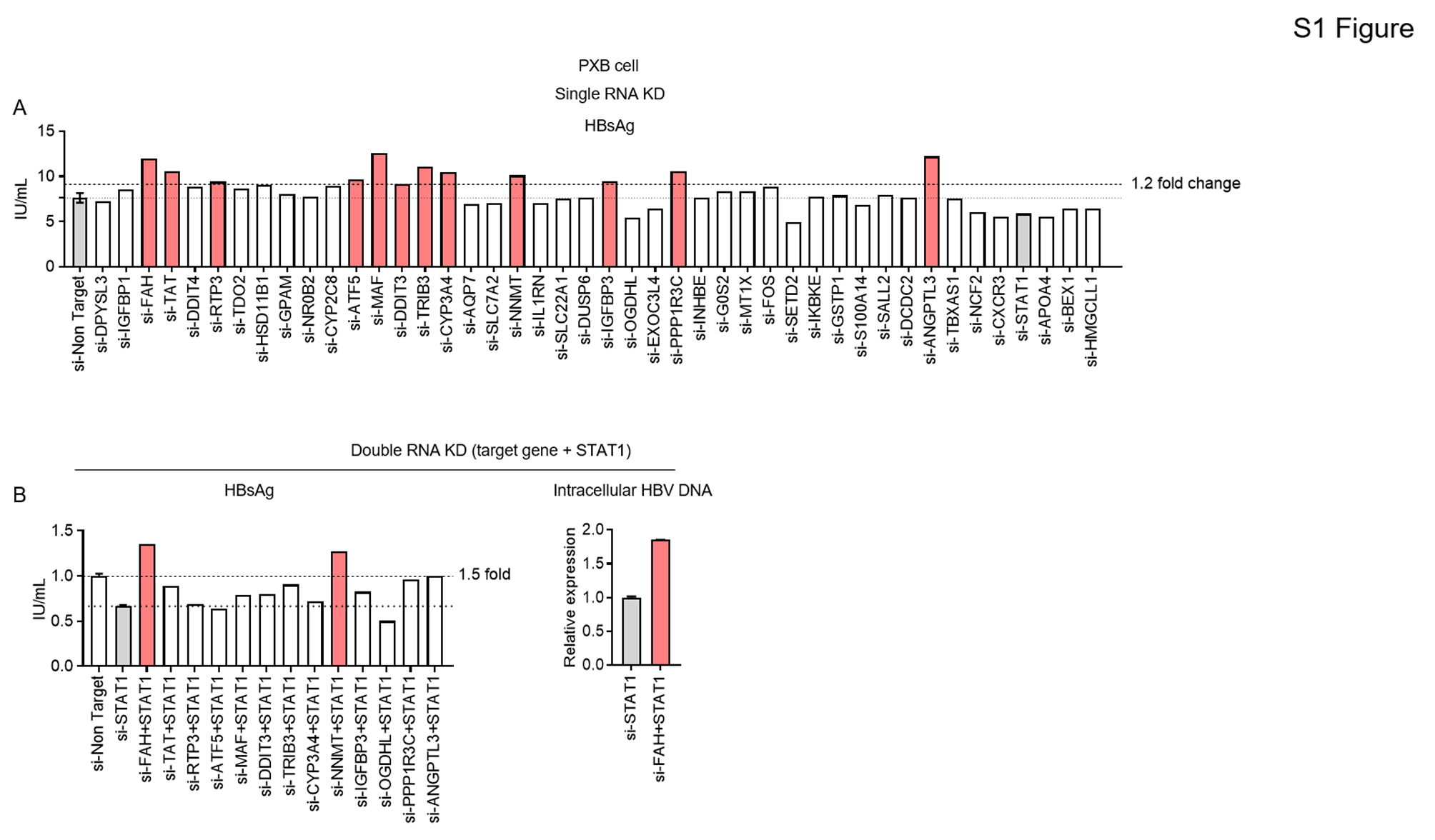

Supplement: S1 Fig — (A) siRNA of 43 candidate genes from the Cherry-pick library was transfected into the PXB cells on day 0 and HBV was added on day 4. The extracellular hepatitis B surface antigen (HBsAg) was analyzed on day 13. The culture medium was exchanged on days 4, 6, and 7. The 1.2-fold value of HBsAg in the sample transfected with the si-Non-Target is indicated by the dotted line. (B) Double siRNA of 12 target genes and STAT1 was transfected into the PXB cells on day 0 and HBV was added on day 4. The extracellular HBsAg and intracellular HBV DNA was analyzed on day 13. The 1.5-fold value of HBsAg in samples transfected with si-STAT1 is indicated by the dotted line. Abbreviations: HBsAg, hepatitis B surface antigen; HBV, hepatitis B virus; PXB, primary human hepatocyte; siRNA, small interfering RNA. (TIF) [file pone.0314581.s001.tif]

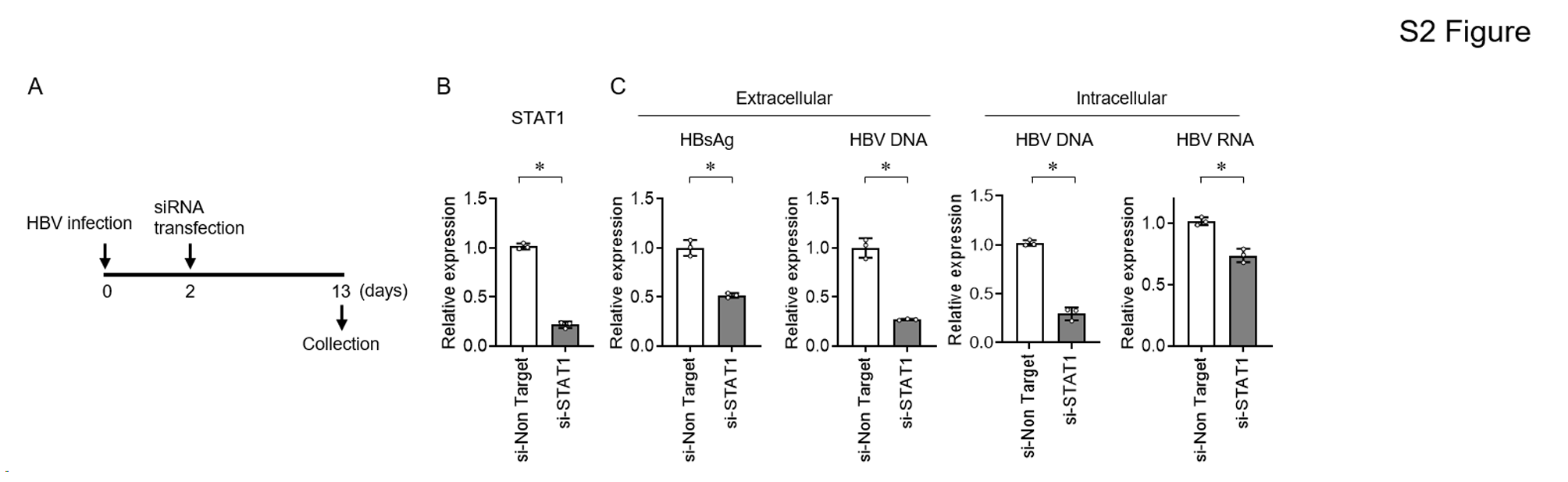

Supplement: S2 Fig — (A) HBV was added on day 0 and siRNA was transfected into the PXB cells on day 2. The supernatant and PXB cells were collected on day 13. The culture medium was exchanged on day 2, 4, and 7. (B) mRNA expression of STAT1 and (C) extracellular HBsAg and HBV DNA and intracellular HBV DNA and HBV RNA were analyzed on day 13. (TIF) [file pone.0314581.s002.tif]

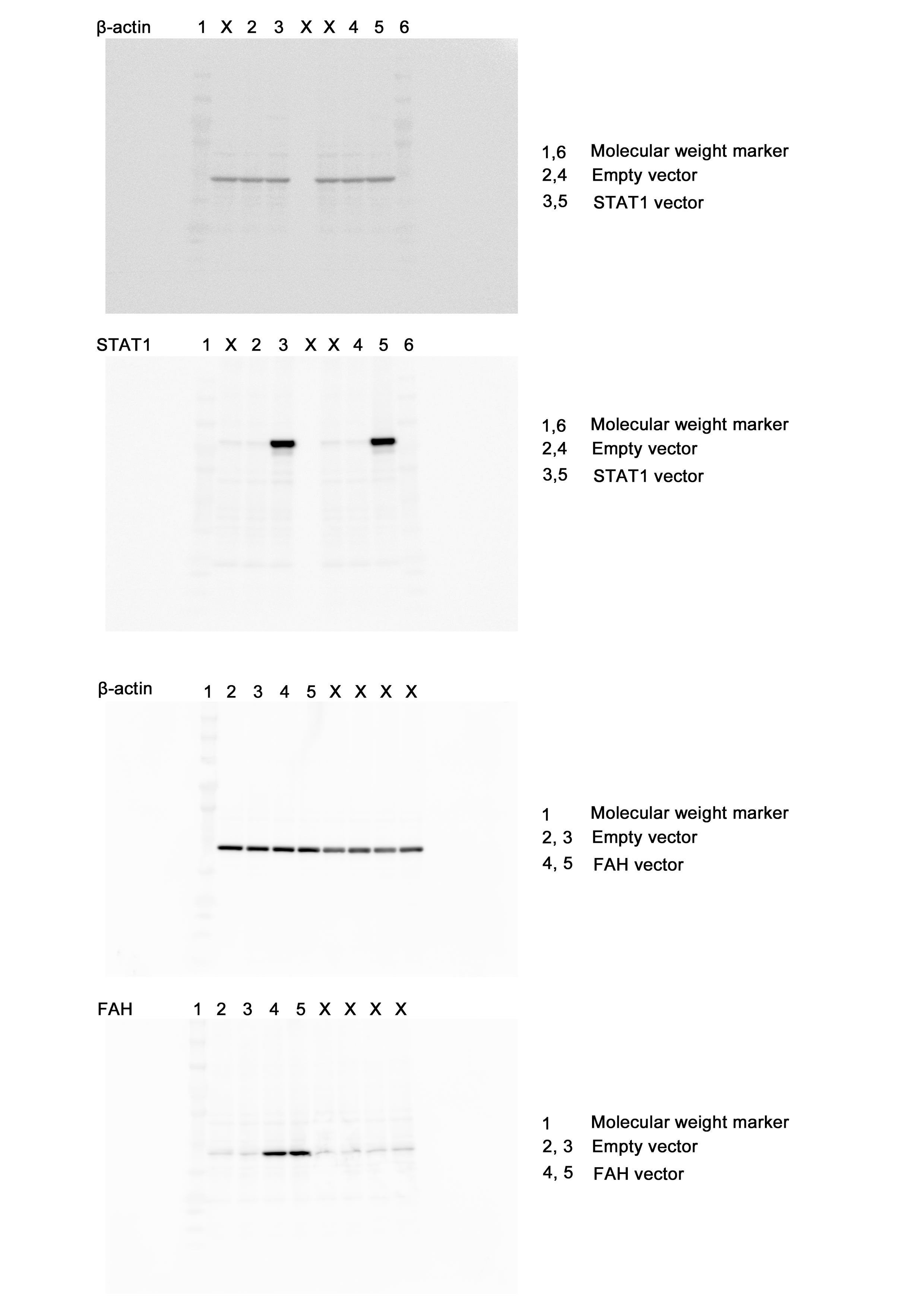

Supplement: S3 Fig — (TIF) [file pone.0314581.s003.tif]
