# Supplementary material for: Identification of novel antiviral host factors by functional gene expression analysis using in vitro HBV infection assay systems
Source: PLoS One. 2025 Mar 6;20(3):e0314581. doi: 10.1371/journal.pone.0314581 (PMC11884705; doi:10.1371/journal.pone.0314581)
Supplement: Attachment — Submitted filename: Response to Reviewers; PONE-D-24-31113R1.docx [file pone.0314581.s004.docx]

November 7, 2024

Dr. Youkyung H. Choi, Ph.D.

Academic Editor

*PLOS ONE*

RE: PONE-D-24-31113R1

“Identification of novel antiviral host factors by functional gene expression analysis using *in vitro* HBV infection assay systems” by T. Nosaka *et al*.

Dear Dr. Youkyung H. Choi:

We sincerely appreciate your thoughtful comments on our above-mentioned manuscript. We thoroughly read the comments and modified the manuscript accordingly as described herein. We have carefully revised the manuscript according to the comments as much as possible. We have included our point-by-point replies to the reviewers’ comments below.

We hope that you will find the revised manuscript to be sufficiently improved to be acceptable for publication in *PLOS ONE*.

Thank you for your reconsideration.

Sincerely,

Yasunari Nakamoto, M.D., Ph.D.

Professor and Head,

University of Fukui, Faculty of Medical Sciences,

Second Department of Internal Medicine,

23-3 Matsuoka Shimoaizuki, Eiheiji-cho, Yoshida-gun,

Fukui 910-1193, Japan.

Tel: +81-776-61-8351, Fax: +81-776-61-8110,

E-mail: nakamoto-med2@med.u-fukui.ac.jp

**Reviewer #1:**

**The revised manuscript improved to make better understanding. However, a few things need to be corrected. Figures 1, 3, and S1 still contain plural descriptions in graph description and line labeling. A reference in line 164 needs to be formatted as other ones.**

We appreciate your important suggestion. We revised plural descriptions in graph description and line labeling in Figures 1, 3, and S1. We formatted a reference in line 164 as other ones.
